# Construction of multi-robot platform based on dobot robots

**DOI:** 10.3389/fnbot.2025.1550787

**Published:** 2025-02-05

**Authors:** Jinchi Han, Duojicairang Ma

**Affiliations:** ^1^School of Professional Studies, Columbia University, New York, NY, United States; ^2^School of Mechanical and Electronic Engineering, East China University of Technology, Nanchang, China

**Keywords:** distributed control, robot kinematics, multirobot systems, simulations and physical experiments, multi-robot platform

## Abstract

For the researches of cooperative control scheme for multirobot systems, this paper sets up an experimental platform based on dobot robots, which can be used to perform physical experiments to verify related schemes. A distributed scheme is proposed to achieve cooperative control for multirobot systems. Simulation results prove the effectiveness of the distributed scheme. Then, the experimental platform based on dobot robots is built to verify the proposed scheme. Specifically, a computer sends data to the microcontroller inside the host through WiFi communication, then the host distributes data to the slaves. Finally, the physical experiment of related schemes is performed on the experimental platform. Comparing the simulations with the physical experiments, the task is successfully completed on this experimental platform, which proves the effectiveness of the scheme and the feasibility of the platform. The experimental platform developed in this paper possesses the capability to validate various schemes and exhibits strong expandability and practicality.

## 1 Introduction

Physical experiments (Xie et al., 2024b; Liufu et al., 2024; Liu et al., 2024; Jin et al., 2021) have always been a crucial part of scientific researches. Induction logging in an inclined fractured formation (Nan et al., 2022) is studied through numerical simulations and physical experiments. However, few experimental platforms can combine simulations with physical experiments. This is negative for the development of relevant researches. To make the results of simulations be objective and accurate, we need simulations and physical experiments to confirm each other, so researchers need a highly adaptable, efficient, and stable platform to verify the developed schemes. In this regard, constructing an experimental platform represents an effective strategy to enhance this scenario.

In recent years, robotics has been rapidly developed in the field of engineering, which is widely used in many areas (Jin et al., 2024b; Xie and Jin, 2024; Jin et al., 2024a; Zhang et al., 2025; Xie et al., 2024a; Ma et al., 2024). For example, the potential of robotics (Xia et al., 2024) in medicine is analyzed. A wall climbing robot (Tago et al., 2024) that can climb vertical walls is introduced to carry out these dangerous tasks. To solve the problem of robots with unknown structures, a scheme (Xie et al., 2024c) is designed, which also considers the avoidance of obstacles. A fuzzy neural controller (Zhang et al., 2023a) for cooperative control of multirobot system is studied. A distributed cooperative control strategy (Zhang et al., 2023b) is designed, which has multiple optimization objectives. Robot control is generally based on the kinematics (Yan et al., 2024; Chen et al., 2024), Denavit–Hartenberg (D–H) parameters of robots (Corke, 2007) are crucial for kinematic control. For example, the processes of kinematic solution (Tang et al., 2024; Xiao et al., 2017; Liao et al., 2022) based on the D–H parameters are described. For multirobot systems, there are three primary control methods: centralized method (Milutinović and Lima, 2006), decentralized one (Draganjac et al., 2016), and distributed one (Zhang et al., 2020). Compared with other methods, distributed one (Jin et al., 2024c) does not require global communication for information exchange but only needs neighbor-to-neighbor communication, which increases the efficiency and stability of systems. Theories for cooperative control of multirobot systems (Zhang et al., 2022; Jin et al., 2021; Zheng et al., 2024) are developing rapidly, but the researches on physical experimental platforms that can verify their practical effects are inadequate. So far, there are few experimental platforms to realize the corresponding physical experiments for distributed cooperative control of multirobot systems, and the experimental platform based on the dobot robots has not been built.

**Figure 1:**
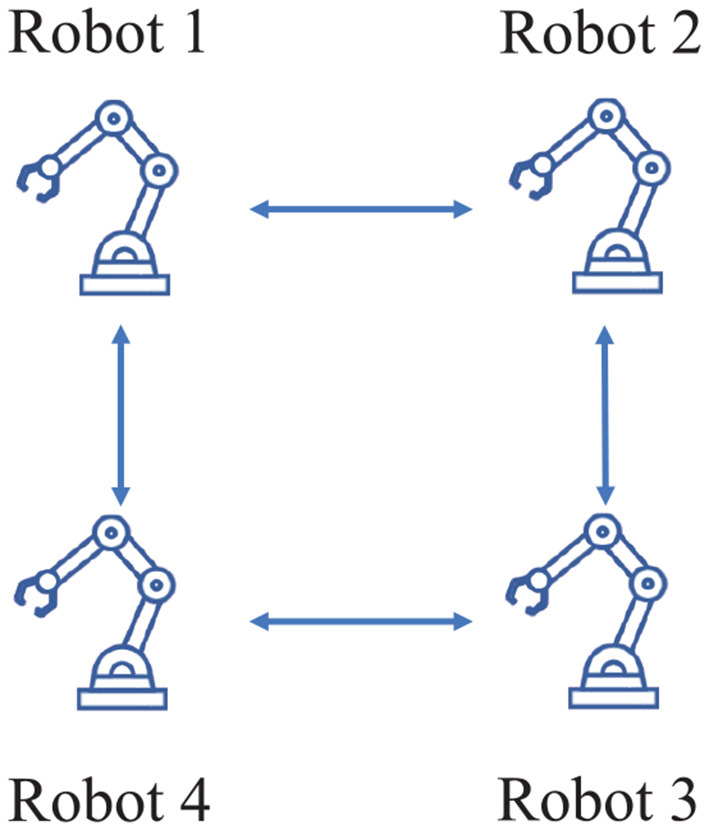
Network topology in the manuscript.

In this paper, the transformation matrix and Jacobian matrix are obtained based on the D–H parameters of the dobot robot, then a distributed scheme (Jin et al., 2018) is employed to verify the effectiveness of the platform. The network topology is restricted to connected and undirected configuration, which is shown in Figure 1. The robots interact with each other via WiFi communication, which is achieved through ESP8266 communication module. Simulations and physical experiments are combined to build the whole platform successfully.

The rest of this paper is divided into four sections. Section 2 introduces the modeling and parameter conventions of the robot, and describes the related formulas in the simulation experiment. It is introduced the hardware and simulation results of the platform in Section 3. Section 4 describes the operation and debugging of the platform. The summarization of the whole paper is given in Section 5. Before ending this section, important contributions which this work achieves are introduced as follows:

1. A multirobot platform based on dobot robots is built to verify cooperative control schemes.
2. Simulation experiments and physical experiments based on multi-dobot platform demonstrate the effectiveness of the experimental platform.

## 2 Preliminaries

**Figure 2:**
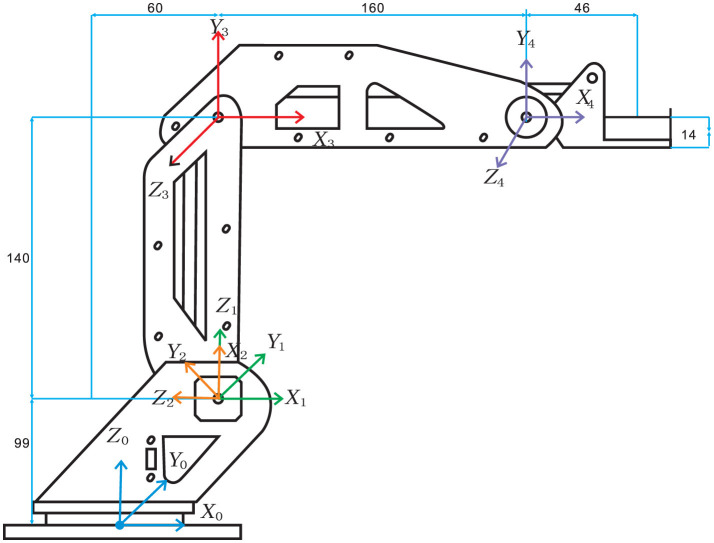
Diagram of joint coordinate system for the dobot robot.

In this part, a kinematic analysis for a single dobot robot is carried out, whose end-effector position is uniquely determined by its configuration in joint space. As shown in Figure 2, a dobot robot with three degrees of freedom is taken as an example. Three joints connect four links (the last link is an end effector) in series.

### 2.1 Establishment of Joint Coordinate System

The dobot robot is mainly composed of a rotating body, a big arm, a small arm, and an end-effecter. It is a typical operating robot, which has three revolute joints to complete the clamping, writing, welding, handling, carving, and other work. In order to describe the relative position and direction relationship among the robot links, it is necessary to establish a coordinate system on each link according to the joint structure. According to the D–H convention (Siciliano and Khatib, 2016), the coordinate system is constructed as Figure 2. The D–H parameters of the dobot robot are listed in Table 1. Baesd on the D–H transformation rule (Siciliano and Khatib, 2016), the transformation matrix is obtained as follows:


Tii−1=​[c(θi)​​−s(θi)​​0​​ai−1​​s(θi)c(αi−1)​​c(θi)c(αi−1)​​−s(αi−1)​​−dis(αi−1)​​s(θi)s(αi−1)​​c(θi)s(αi−1)​​c(αi−1)​​dic(αi−1)​​0​​0​​0​​1​],

where c(θ_*i*_) = cos(θ_*i*_), s(θ_*i*_) = sin(θ_*i*_), c(α_*i*_) = cos(α_*i*_), s(α_*i*_) = sin(α_*i*_).

**Table 1:** D–H parameters of the dobot robot.

***i***	**α_*i*−1_/(rad)**	***a*_*i*−1_/(m)**	***d*_*i*_/(m)**	**θ_*i*_/(rad)**
1	0	0	0.097	θ_1_
2	−π/2	0	0	θ_2_
3	0	0.14	0	θ_3_
4	0	0.16	0	θ_4_

### 2.2 Robot kinematics

**Figure 3:**
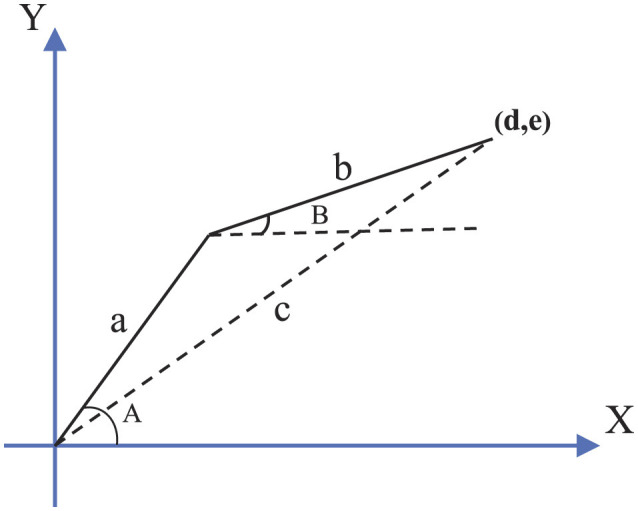
Diagram of kinematics coordinate system for the dobot robot.

Given a fixed trajectory $r_{\text{c}}\left(t\right)\in {\mathbb{R}}^{p}$ of the end-effector, we need to generate **φ**(*t*) = [**φ**_1_(*t*), **φ**_2_(*t*), ⋯ , **φ**_*q*_(*t*)]^T^ ∈ ℝ^*q*^ to control the robot. It should be noted here that the Cartesian coordinate ***r*** ∈ ℝ^*p*^ in the workspace of a robot is uniquely determined by a nonlinear mapping:


(1) $$\begin{array}{l} r\left(t\right)=\hbar \left(\varphi \left(t\right)\right), \end{array}$$

where ℏ(·) is a nonlinear differentiable function for a given robot with known parameters. Calculating the time derivatives of both sides of Equation 1, a kinematic equation for the joint velocity level is shown below:


(2) $$\begin{array}{l} \dot{r}\left(t\right)=J\left(\varphi \left(t\right)\right)\dot{\varphi }\left(t\right), \end{array}$$

where Jacobian matrix *J*(**φ**(*t*)) = ∂ℏ/∂**φ** ∈ ℝ^*p*×*q*^ is generally abbreviated as *J*. The inverse kinematics problem of a robot is to determine joint variables of the robot based on the given position of the end-effector, which is shown in Figure 3. The relations between the given position of the end-effector and joint angles of the dobot robot are:


(3) $$\begin{array}{l} A=\arccos\frac{a^{2}+d^{2}+e^{2}-b^{2}}{2a\sqrt{d^{2}+e^{2}}}, \end{array}$$

(4) $$\begin{array}{l} B=\arccos\frac{a^{2}+b^{2}-d^{2}-e^{2}}{2ab}+A-\pi . \end{array}$$

The expressions for the joint angles are introduced as


(5) $$\begin{array}{l} A=\arccos\frac{a^{2}+c^{2}-b^{2}}{2ac}, \end{array}$$

(6) $$\begin{array}{l} B=\arccos\frac{a^{2}+b^{2}-c^{2}}{2ab}+A-\pi . \end{array}$$

## 3 Hardware and simulation

This section describes the dobot robot, which is employed to build the physical platform. Details of the physical platform are also introduced and presented. In addition, the results of the simulation experiments are also described.

### 3.1 Dobot

**Figure 4:**
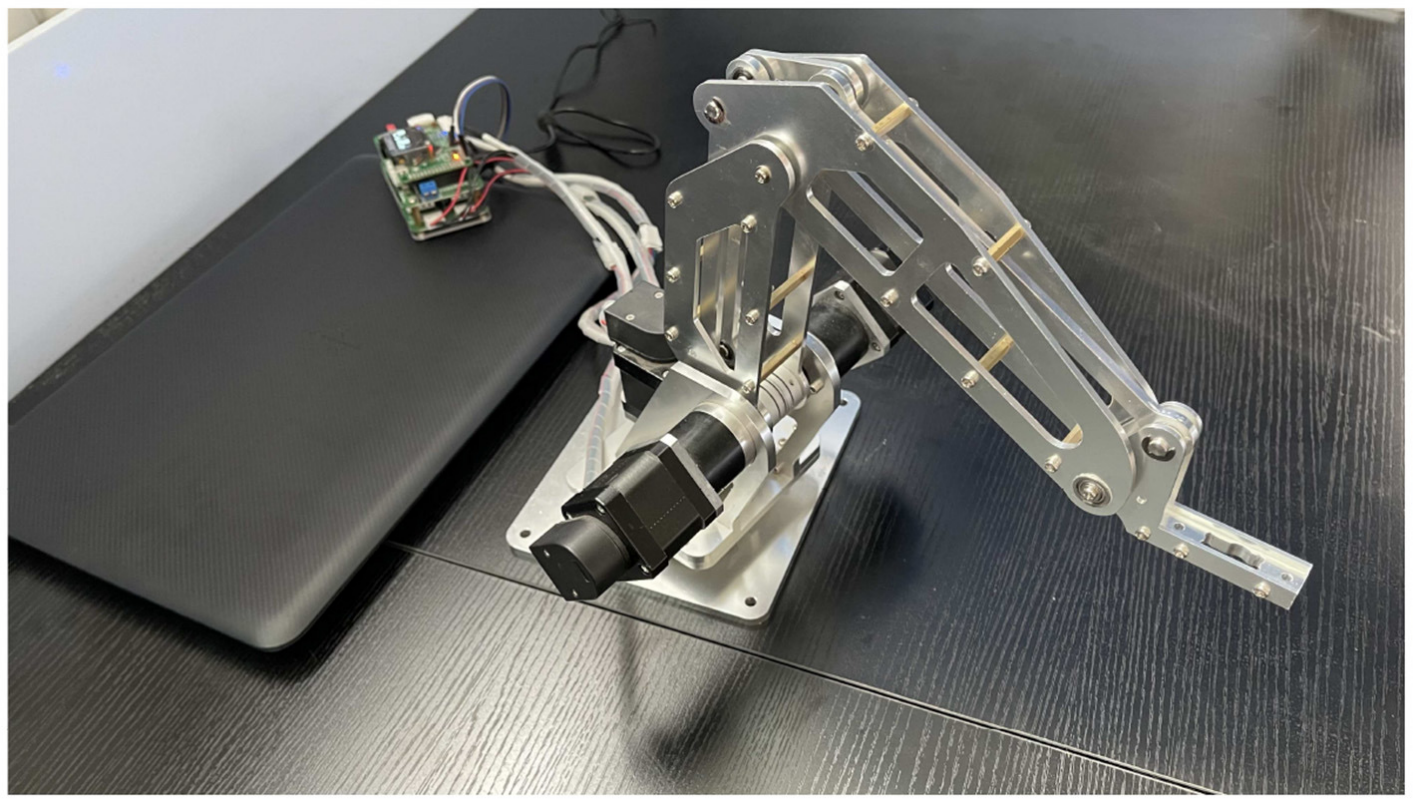
The dobot robot.

The robot selected for the physical platform is the dobot robot, which is shown in Figure 4. The mechanical structure of the dobot robot is composed of a rotating base, a large arm, a small arm, and an end-effector. The STM32 microcontroller governs the rotation of three motors to facilitate the operation of the dobot robot.

**Figure 5:**
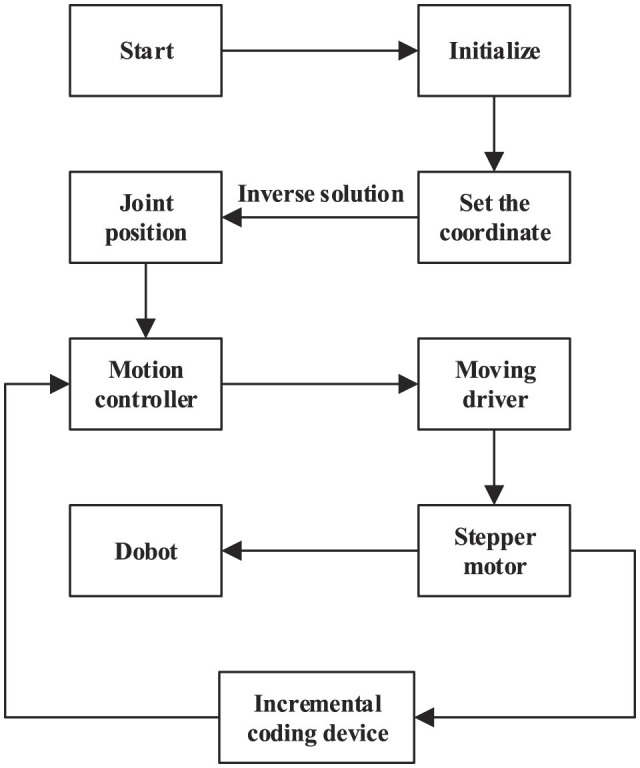
System flow chart.

After solving the inverse kinematics of the dobot robot, the expression between the coordinates of the end-effector and the joint angle is obtained. According to the expression, the dobot robot is controlled by a single-chip computer. Figure 5 is the flow chart of the single-chip computer program.

**Figure 6:**
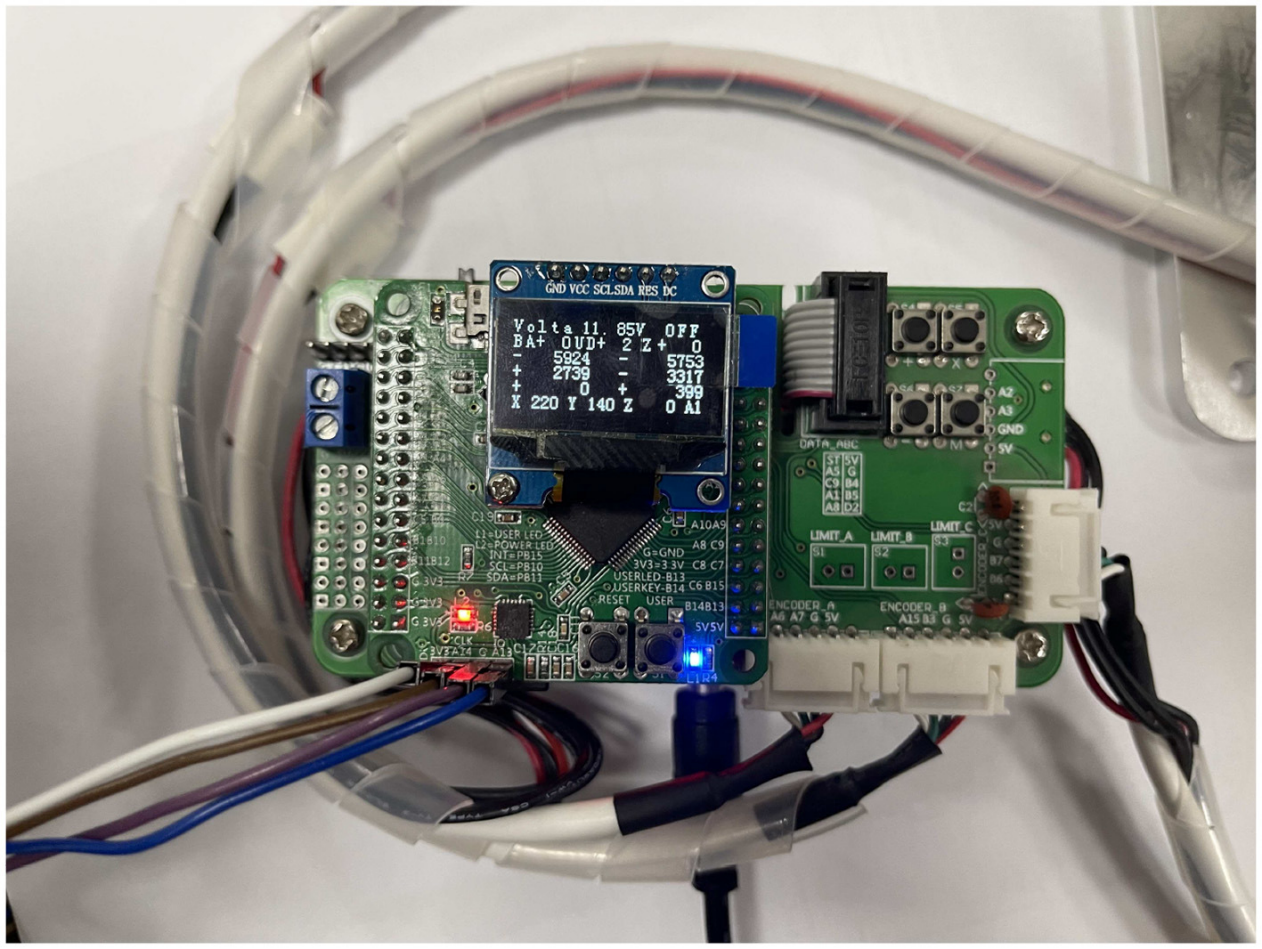
Real-time coordinate display.

In order to achieve cooperative control of multirobot systems, actual coordinates of end-effectors have to be shared online, so we add incremental encoders to record coordinates of end-effectors. The operation of the dobot robot motor is converted into digital signals by the encoder so that these digital signals can be sent to the next microcontroller of dobot to achieve cooperative control. Figure 6 shows the status information of the dobot robot obtained by the encoder, in which the actual position of the end-effector is available.

The stepper motor used in dobot robot is the typical 42 mm stepper motor. Each joint is controlled by a motor. The stepping angle of the motor is 1.8 degrees. 200 pulses are required to rotate the motor rotor 360 degrees in full-step mode. Due to the addition of a reducer on the stepper motor, the reduction ratio is 1:10, so the rotor actually turns a circle needs 2,000 pulses, a pulse turns 0.18 degrees. To precisely control the dobot robot to rotate through a fixed angle, it is necessary to set a fixed initial position and check the initial position when the dobot robot is powered on and initialization. This ensures that the joint angle of the dobot robot corresponds to the value of joint angle in the variable.

### 3.2 Physical experimental platform

The physical experimental platform consists of a computer and four dobot robots. A computer verifies the scheme of robotic cooperative control through simulation, then the data obtained is transmitted to the host machine, which sends the data to the rest of the slaves. Each robot starts to execute the program, so that the physical experiment to verify the scheme can be completed.

Because user datagram protocol (UDP) (Rutkowski, 2016; Pai et al., 2024) can realize broadcast communication with low delay and high transmission efficiency, we use connectionless UDP to realize data transmission among dobot robots. The first dobot robot is set as the master and the rest dobot robots are developed as slaves. A computer is used to send data to the STM32 microcontroller (Ktari et al., 2023; Guo et al., 2019; Li et al., 2020), which is in the dobot robot via universal asynchronous receiver/transmitter. The STM32 microcontroller controls wireless communication module ESP8266 (Amaral-Júnior et al., 2019; Wiryasaputra et al., 2023; Siam et al., 2023) by AT commands to send and receive data, thus realizing cooperative control. The communication structure of the physical experimental platform consists of three parts: the upper computer, the host, and the slaves. When verifying the scheme, the upper computer generates the data after the simulation is conducted, which is send to the STM32 through the serial port. The STM32 of the host receives the data and starts to execute the action, and then controls the ESP8266 module to send the control data to the slaves via WiFi. The slaves receive the data and check with the host and start to execute the action.

### 3.3 Simulation result

**Figure 7:**
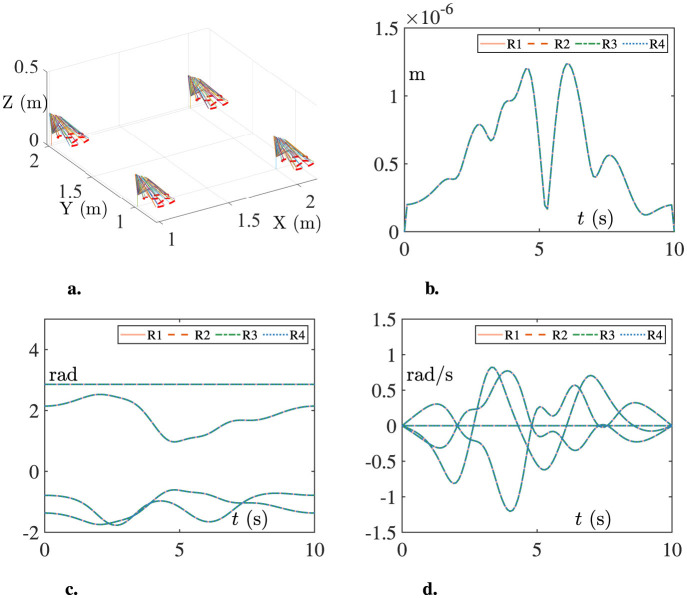
Simulation results of the multi-robot platform tracking the Lissajous-curve. **(A)** The three-dimensional view. **(B)** The tracking errors. **(C)** The joint angles. **(D)** The joint velocities.

Based on parameters of the dobot robot and distributed scheme (Jin et al., 2018), the simulation results are shown in Figure 7. The three-dimensional view of the experimental platform in action is shown in Figure 7A, which shows that the trajectory tracking task is successfully completed. The variation of tracking errors for end-effectors is shown in Figure 7B. The errors maintain at about 10^–6^ m after starting operation. Besides, the variations of joint angles and joint velocities are shown in Figures 7C, D.

## 4 Physical experiments

In this section, we present the outcomes of the physical experiments conducted on this platform. Furthermore, we provide an overview of the debugging process for the physical experiment platform.

### 4.1 Physical experiments based on simulation result

**Figure 8:**
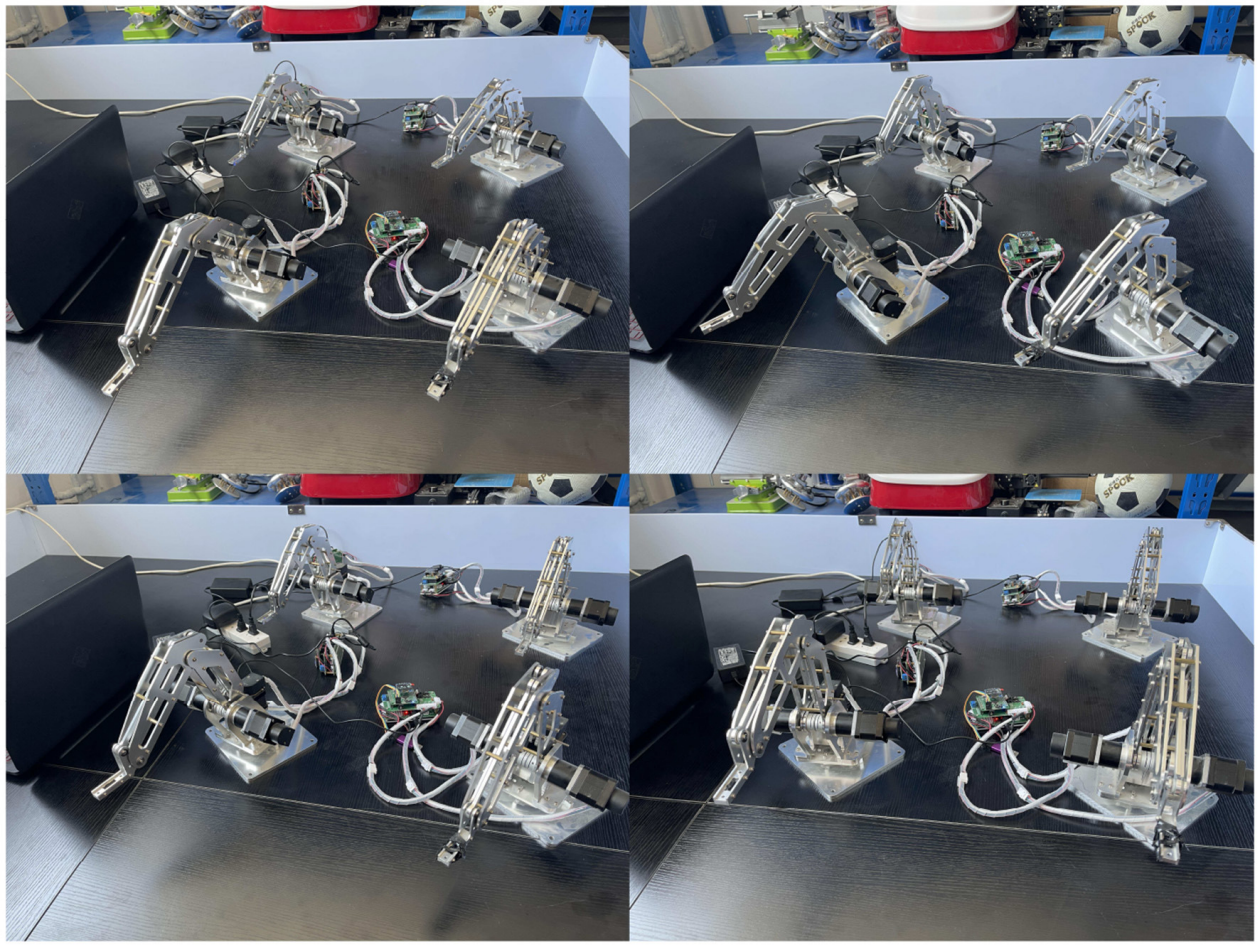
Snapshots of the physical experimental platform utilized to validate the distributed scheme (Jin et al., 2018).

Snapshots illustrating the physical experimental platform utilized to validate the distributed scheme (Jin et al., 2018) is presented in Figure 8. First, we input commands to the master, and then the master transmit the commands to the remaining three slaves through WiFi communication, thus achieving cooperative control. After testing and debugging, the platform is successfully constructed, which is able to be used to verify various schemes of cooperative control for multirobot systems.

### 4.2 Platform debugging

The data obtained from simulations is transmitted to the host by WiFi communication, and then the host distributes data to the slaves. In the process of conducting physical experiments, we encounter the following problems:

1. If there is a discrepancy in the start positions of dobot robots, it may cause the dobot robots to fail to reach the target position.
2. Packet loss can occur during data transmission, which leads to inconsistent trajectories between the slave and the host.
3. The actual joint angles of dobot robots are different from the data collected by the encoder, resulting in inconsistency between the actual trajectory and the simulated trajectory.

We analyze the causes and make targeted improvements based on the problems that occur during physical experiments. The problem is finally solved by the following methods:

1. In the STM32 microcontroller, a fixed coordinate is added after the initialization procedure, which allows dobot robots to start verifying when they reach the fixed initial positions. This ensures that each dobot robot initiates from an identical starting position.
2. Packet loss is generated because the frequency of sending data is too high. After setting the socket receive cache, packet loss is greatly reduced. This makes all dobot robots in the physical experimental platform to track the same trajectory.
3. The errors between the collected data and the actual data are attributed to the malfunctions of stepper motors, which are solved after replacing stepper motors.

It is worth mentioning that the dobot robot possesses inherent hardware limitations, notably the precision of its stepper motor. While the addition of encoders can enhance accuracy to some extent, the fundamental hardware performance of the robot continues to influence the experimental outcomes. Besides, although the UDP has the advantages of low latency and high efficiency, but in the actual experiment, data packet loss and delay may become the key factors affecting the experimental results.

## 5 Conclusion

This paper has proposed a physical experimental platform for the cooperative control of multirobot systems. The comparison of simulations and physical experiments has been performed to substantiate the superiority, effectiveness, and accuracy of the physical experimental platform. For the first time, the experimental platform is able to realize the simulations and physical experiments simultaneously to verify the cooperative control scheme of multi-robot systems. In addition to the synchronous verification scheme, UDP is used to send and receive data via WiFi communication in the experimental platform designed in this paper, which significantly enhances the usefulness of the platform. As the other research hotspot in distributed control of multi-robot systems, it is imperative to improve the impact of time delays on the stability of systems, as practical systems are inherently causal and thus subject to varying degrees of time delays. Consequently, this research topic can be treated as a promising future direction. In addition, a collaborative experimental control platform based on redundant robots would be a potential direction for future researches.

## Data Availability

The raw data supporting the conclusions of this article will be made available by the authors, without undue reservation.
